# Structure Characterization and Hypoglycaemic Activities of Two Polysaccharides from *Inonotus obliquus*

**DOI:** 10.3390/molecules23081948

**Published:** 2018-08-04

**Authors:** Ping Liu, Jiao Xue, Shisheng Tong, Wenxia Dong, Peipei Wu

**Affiliations:** 1 College of Food Science and Nutritional Engineering, China Agricultural University, Beijing 100083, China; xj1154009143@163.com (J.X.); shuofangssdd@163.com (P.W.); 2 Bio-Pharmaceutical College, Beijing City University, Beijing 100094, China; shishengt@163.com (S.T.); 3 College of Life Sciences, Qufu Normal University, Jining 273165, China; dongwenxia2018@163.com (W.D.)

**Keywords:** *Inonotus obliquus*, polysaccharide, structure, α-glucosidase inhibitory activity, HepG2 cells

## Abstract

In the present study, two polysaccharides (HIOP1-S and HIOP2-S) were isolated and purified from *Inonotus obliquus* using DEAE-52 cellulose and Sephadex G-100 column chromatography. The structural characterization and in vitro and in vivo hypoglycaemic activities of these molecules were investigated. HPLC analysis HIOP1-S was a heterpolysaccharide with glucose and galactose as the main compontent monosaccharides (50.247%, molar percentages). However, HIOP2-S was a heterpolysaccharide with glucose as the main monosaccharide (49.881%, molar percentages). The average molecular weights of HIOP1-S and HIOP2-S were 13.6 KDa and 15.2 KDa, respectively. The β-type glycosidic bond in HIOP1-S and HIOP2-S was determined using infrared analysis. ^1^H-NMR spectra indicated that HIOP2-S contains the β-configuration glycosidic bond, and the glycoside bonds of HIOP1-S are both α-type and β-type. The ultraviolet scanning showed that both HIOP1-S and HIOP2-S contained a certain amount of binding protein. Congo red test showed that HIOP1-S and HIOP2-S could form a regular ordered triple helix structure in the neutral and weakly alkaline range. HIOP1-S and HIOP2-S showed strong α-glucosidase inhibitory activities and increased the glucose consumption of HepG2 cells. In addition, Streptozotocin (STZ)-induced hyperglycaemic mice were used to evaluate the antihyperglycaemic effects of HIOP1-S and HIOP2-S in vivo. The results showed that HIOP2-S had antihyperglycaemic effects. Taken together, these results suggest that HIOP1-S and HIOP2-S have potential anti-diabetic effects.

## 1. Introduction

Diabetes mellitus (DM) is a chronic metabolic disorder caused by insulin deficiency or functional disturbance of the receptors. Diabetes mellitus has become a worldwide epidemic, with the number of diabetes mellitus patients increasing annually. In 2013, 382 million individuals worldwide suffered from diabetes, and the total number of individuals with diabetes is expected to reach 592 million by 2035 [1]. Currently, the effective anti-diabetic medicines in the pharmaceutical market include α-glucosidase inhibitors, biguanides and sulphonylureas. However, these frequently used synthetic anti-diabetic drugs are associated with adverse effects and even toxicity after long-term use [2,3]. The potential adverse effects of these anti-diabetic drugs include weight gain, stomach upset, lactic acidosis, swelling of legs or ankles, etc. [4,5]. Thus, there is an urgent need to develop new medications with lower side effects for diabetes.

*Inonotus obliquus* (Chaga) is a rare edible fungus that is parasitic on birch trees, belonging to the family Hymenochaetaceae of Basidiomycetes. Chaga is a typical tree fungus mainly distributed in cold regions of 45° N to 50° N latitude, such as in Northern Russia, Europe, North America and Hokkaido, Japan. *I. obliquus* has been documented to contain poly-saccharides, polyphenols, triterpenes, melanin and steroid, showing various biological activities [6].

Among these main components, polysaccharides, one of the most important compounds, have attracted more and more research interest in recent years. Both domestic and foreign studies have shown that the polysaccharides isolated from *I. obliquus* have distinct immunomodulation [7,8]. antitumour [9,10] and antioxidant capacities [11,12,13]. However, to date, no detailed studies characterizing the composition and hypoglycaemic capacity of different polysaccharides isolated from *I. obliquus* have been reported. Therefore, the aims of the present study were to purify and characterize the properties of different polysaccharides fractions from *I. obliquus* and explore the hypoglycaemic activities of these compounds to identify new biological functional components for use in the pharmaceutical industry.

## 2. Results

### 2.1. Homogeneity and Molecular Weight of HIOP1-S and HIOP2-S

**Figure 1:**
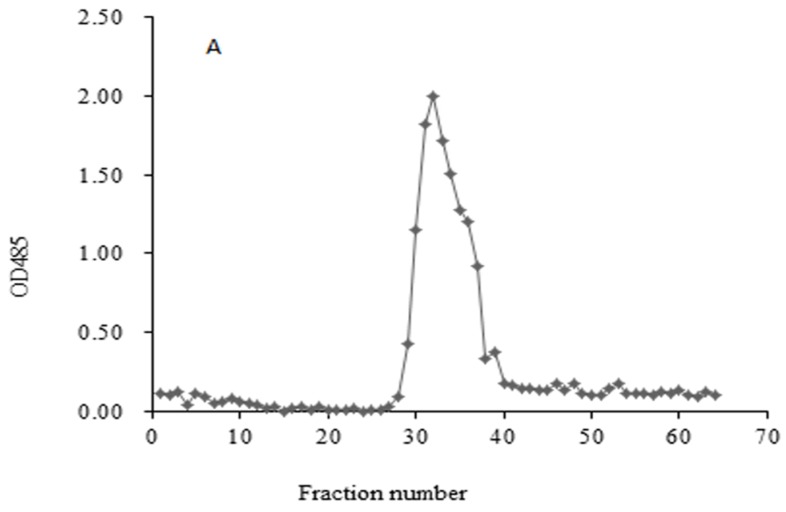

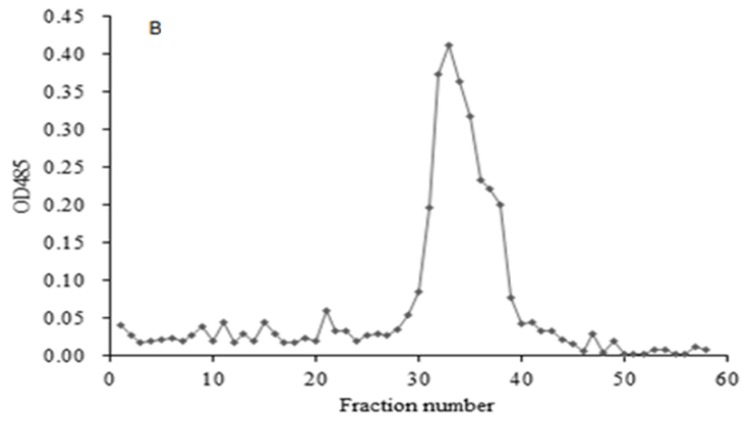
(**A**) Sephadex G-100 column chromatography for HIOP1-S; (**B**) Sephadex G-100 column chromatography for HIOP2-S.

**Figure 2:**
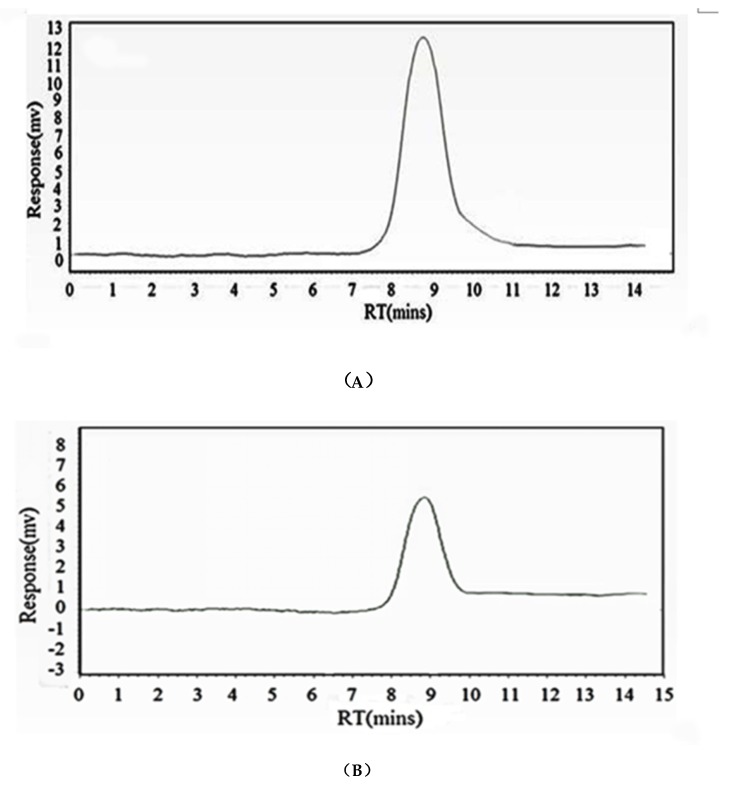
(**A**) The GPC gel chromatography spectrum of HIOP1-S; (**B**) The GPC gel chromatography spectrum of HIOP2-S.

HIOP1-S and HIOP2-S were purified using Sephadex G-100, showing a main peak, detected using a phenol-sulphuric acid assay (Figure 1). As shown in Figure 2, both HIOP1-S and HIOP2-S showed only one single and symmetrical narrow peak with elution time in gel permeation chromatography (GPC), indicating that HIOP1-S and HIOP2-S were polysaccharides with a relatively concentrated molecular weight distribution, consistent with the results of Sephadex G-100 of HIOP1-S and HIOP2-S. Based on the calibration with standard dextrans, the estimated average molecular weights of HIOP1-S and HIOP2-S were 13.6 KDa and 15.2 KDa respectively.

### 2.2. Monosaccharide Composition of HIOP1-S and HIOP2-S

The properties of HIOP1-S, and HIOP2-S, including carbohydrate, protein and uronic acid contents and percentage of monosaccharide components, are summarized in Table 1. Determining the monosaccharide composition and molar ratio of polysaccharides is important to characterize the bioactivity of these compounds. Glucose, galactose, arabinose and fucose were the major monosaccharides of HIOP1-S. HIOP2-S primarily comprised glucose, galactose and mannose, but not fucose, with the highest glucose content of up to 49.881%. These results demonstrated that the monosaccharide ratios of the two polysaccharides were different, and the monosaccharide compositions of HIOP1-S and HIOP2-S were also different from previous findings [14].

### 2.3. FT-IR of HIOP1-S and HIOP2-S

**Figure 3:**
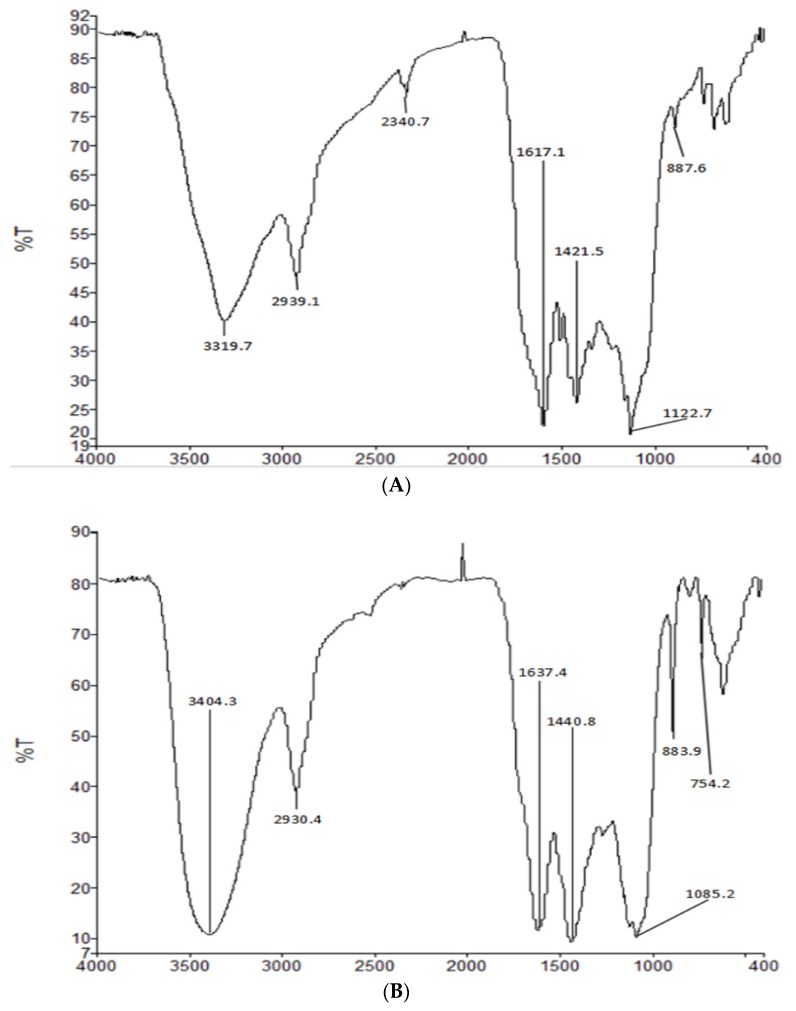
(**A**) Fourier transform infrared spectroscopy of HIOP1-S; (**B**) Fourier transform infrared spectroscopy of HIOP2-S.

IR spectroscopy is a powerful technique for the identification of characteristic organic groups in polysaccharides. As shown in Figure 3, a typical broad stretching peak at approximately 3319.7 cm^−1^ and 3404.5 cm^−1^ in the IR spectra resulted from the stretching vibration of O–H, and the signal at approximately 2939.1cm^−1^ and 2930.4 cm^−1^ resulted from the stretching vibration of C–H [15]. The absorption peaks at approximately 1617 cm^−1^ and 1421 cm^−1^ reflected the presence of carboxyl groups and carbonyl groups [16]. The peaks at approximately 1150 cm^−1^ suggested the presence of a pyranose ring in HIOP1-S and HIOP2-S [17], while characteristic absorption at 887.6 cm^−1^ and 883.7 cm^−1^ indicated the presence of a β-type glycosidic bond in HIOP1-S and HIOP2-S [18]. 

### 2.4. NMR of HIOP1-S and HIOP2-S

At present, the nuclear magnetic resonance method is widely used, and the position of glycosidic bonds, the order of monosaccharide residues, the number of monosaccharides in the repeated structures, and the type of α, β isomers can be determined simultaneously without destroying the polysaccharide structure. The chemical shifts at 4.3–4.9 ppm and 4.9–5.5 ppm in the ^1^H-NMR spectrum could be assigned to the typical signals of the anomeric protons of β- and α-anomers, respectively [19].

**Figure 4:**
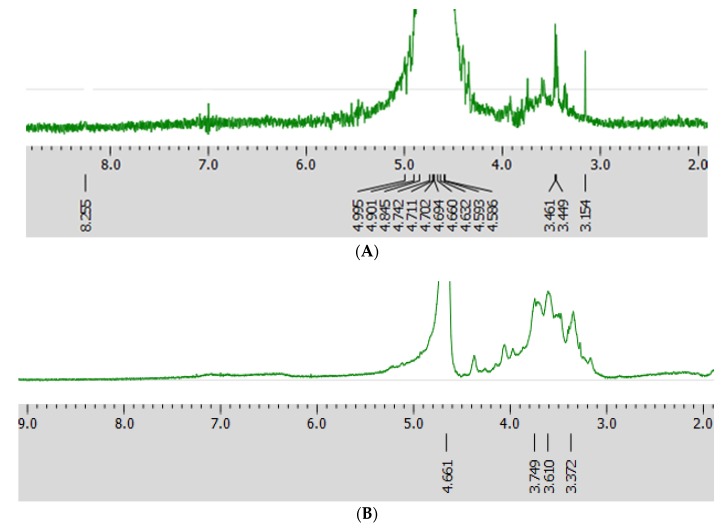
^1^H-NMR spectrum (**A**) HIOP1-S; (**B**) HIOP2-S.

The ^1^H-NMR spectra of HIOP1-S and HIOP2-S are shown in Figure 4. The peak around 4.3 ppm and 5.0 ppm showed HIOP1-S contained α- and β-glycosidic bond. The peak at 4.661 of the chemical shift of HIOP2-S is the proton vibration absorption peak on the sugar ring. There is no absorption peak above 4.9 ppm, indicating that HIOP2-S contains no α-glycosidic bond, weak peak at the 4.4 ppm of HIOP2-S indicated that this polysaccharide contained a small amount of β-glycosidic bonds. The peak at 4.7 ppm was deuterated water. In addition, the analysis suggested that the two polysaccharides were heteropolysaccharides.

### 2.5. Ultraviolet Spectrum Scanning of HIOP1-S and HIOP2-S

**Figure 5:**
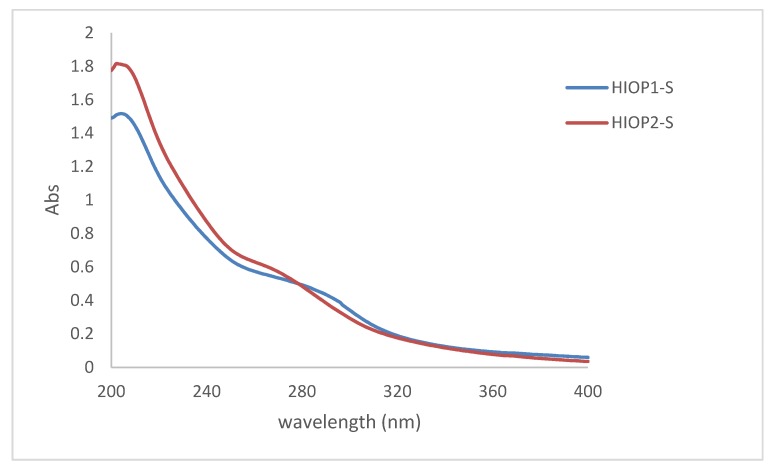
UV absorption spectra of HIOP1-S and HIOP2-S.

The ultraviolet (UV) absorption spectra of the two polysaccharides are shown in Figure 5. As can be seen, two polysaccharides contain ultraviolet absorption peaks of polysaccharides and proteins. Different samples have different absorption peaks at different values at 190 nm, and a lower protein absorption peak appears at around 280 nm. Although the protein is removed by the sevage method, the absorption peak still exists, and it can be speculated that HIOP1-S and HIOP2-S contains a certain amount of binding protein. This is consistent with the previous results.

### 2.6. Congo Red Test of HIOP1-S and HIOP2-S

The conformation of the polysaccharide can be speculated by the Congo red test. Congo red is capable of forming complexes with polysaccharides in a helical conformation and causing a red shift in the maximum absorption wavelength. However, in the presence of a certain concentration of NaOH, hydrogen bonds that maintain the three-stranded helical conformation were destroyed, resulting in a decrease in the maximum absorption wavelength [20].

**Figure 6:**
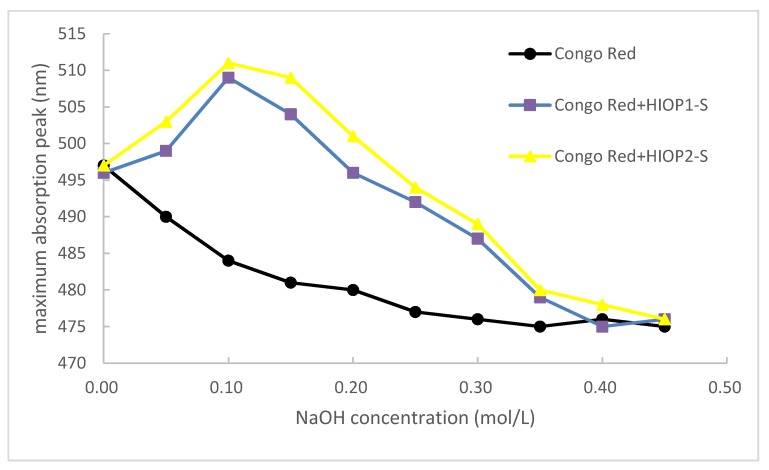
Congro Red test of HIOP1-S and HIOP2-S.

The change of the maximum absorption wavelength of the complex formed by the two polysaccharides with Congo red in the range of NaOH concentration 0–0.45 mol/L was shown in Figure 6. In the concentration range of 0–0.10 mol/L NaOH, UV absorption shift to a long wave, indicating that the polysaccharide and Congo red combine to form a purplish red color, that is, the sample has a regular helical conformation; when it is higher than 0.10 mol/L, the maximum absorption wavelength dropped sharply, indicating that the spiral structure of polysaccharides disintegrated and became irregular. This indicated that HIOP1-S and HIOP2-S can form a regular ordered triple helix in a neutral, weakly alkaline range.

### 2.7. α-Glucosidase Inhibition Activity

The α-glucosidase enzyme, located in the brush border of the small intestine, can hydrolyse the terminal non-reducing α–1 → 4 linkage of oligosaccharides to monosaccharides absorbed by the intestinal epithelia [21]. Therefore, inhibition of α-glucosidase activity can delay the release of glucose in the intestine and reduce postprandial hyperglycemia. Currently, the analysis of α-glucosidase inhibitory activity has been widely used to evaluate the hypoglycaemic activity bioactive compounds in vitro. In this experiment, we used IC50 presented the inhibitory activity.

The inhibitory effects of HIOP1-S, HIOP2-S, and acarbose on the activity of α-glucosidase are shown in Table 2, showing that both HIOP1-S and HIOP2-S had a more significant inhibition effect on α-glucosidase than acarbose. Acarbose is used as an oral hypoglycaemic agent for patients with type 2 diabetes [22]. However, this compound can also lead to intestinal discomfort and other side effects, such as abdominal distension, diarrhoea and meteorism. Many natural polysaccharides exert good inhibitory effects on the activity of α-glucosidase [23,24]. These results indicated that both HIOP1-S and HIOP2-S could be potential inhibitors for α-glucosidase when used in functional foods.

### 2.8. Cytotoxicity of the Polysaccharides at Different Concentrations on HepG2 Cells

**Figure 7:**
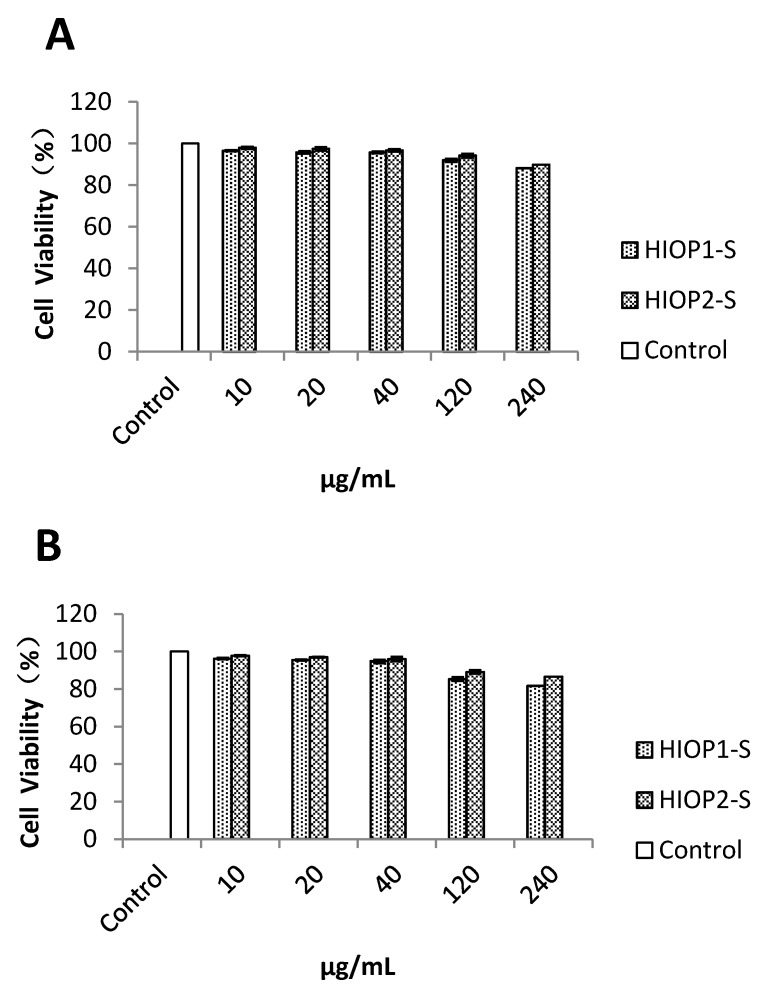
Evaluation of the cytotoxicity of HIOP1-S and HIOP2-S on HepG2 cells (**A**) at 48 h and (**B**) 72 h.

As shown in Figure 7, when the HIOP1-S concentration was higher than 40 mg/mL (after incubation for 48 h), cell viability was lower than 95%, and after incubation for 72 h, cell viability was less than 90%, suggesting that at these concentrations, the drugs show toxic side effects on cells. Under the same conditions, HIOP1-S and HIOP2-S were not cytotoxic to HepG2 cells when incubated for 24 h (data not shown). At 40 mg/mL, the cell viability of HIOP1-S and HIOP2-S was nearly 95%. Therefore, the concentration of 40 mg/mL was not toxic to cells. Finally, 40 mg/mL was set as the high dose, 20 mg/mL was set as the middle dose, and 10 mg/mL was set as the low dose of HIOP1-S and HIOP2-S.

### 2.9. Glucose Consumption Assay

**Figure 8:**
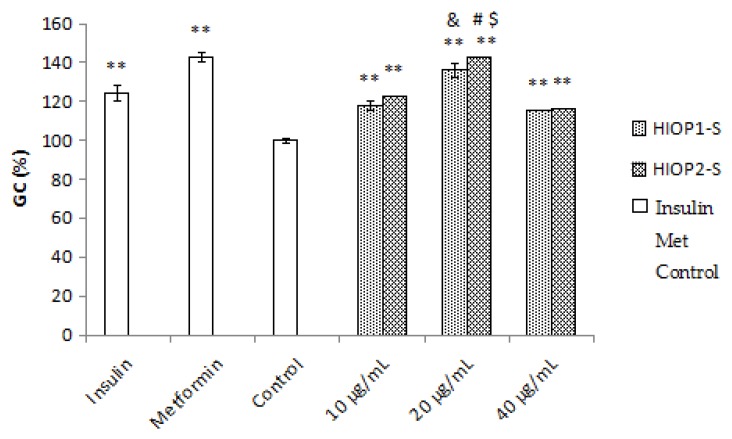
Effects of HIOP1-S and HIOP2-S on the glucose consumption of HepG2 cells. ** *p* < 0.01, DC vs. NC; ^&^ *p* < 0.01, HIOP1-S (20 μg/mL) vs. HIOP1-S (10 and 40 μg/mL); ^#^ *p* < 0.01, HIOP2-S (20 μg/mL) vs. HIOP2-S (10 and 40 μg/mL); and ^$^ *p* < 0.05, HIOP2-S (20 μg/mL) vs. HIOP1-S (20 μg/mL). The data are expressed as the means ± SD.

Insulin resistance is highly correlated with the consumption of extracellular glucose, reflecting decreased sensitivity of insulin receptors. As shown in Figure 8, the exposure of HepG2 cells to 10^−7^ mmol/L of insulin significantly decreased the consumption of extracellular glucose compared with the normal control group (*p* < 0.01), and the positive control metformin (10^−3^ mmol/L) remarkably reversed the glucose consumption to normal levels. Expectedly, treatments with HIOP1-S and HIOP2-S (10, 20 and 40 µg/mL) significantly increased the GC in insulin-resistant HepG2 cells (*p* < 0.01). Interestingly, treatment with HIOP2-S (20 µg/mL) significantly increased extracellular glucose consumption compared with HIOP1-S (20 μg/mL) (*p* < 0.05). These results showed that HIOP1-S and HIOP2-S improve extracellular glucose consumption in insulin-resistant HepG2 cells, and more importantly, HIOP2-S exhibited a stronger effect than HIOP1-S. Studies have reported a polysaccharide extracted from the fruit of *Lycium barbarum* L. with significant hypoglycaemic effects through promoting β cell proliferation and increasing the activities of key enzymes of glucose metabolism [25]. Gong Y, Jie Z, Fei G, et al. reported that three polysaccharides extracted from Maidong can mitigate insulin resistance in HepG2 cells through the PI3K/AKT pathway [26]. Therefore, the underlying mechanisms of the hypoglycaemic effects of HIOP1-S and HIOP2-S require further research. Nevertheless, these data indicated HIOP1-S and HIOP2-S as potential hypoglycaemic agents in the treatment of diabetes.

### 2.10. Effect of HIOP1-S and HIOP2-S on Fasting Blood Glucose in STZ-Induced Diabetic Mice

As shown in Table 3. The initial blood glucose levels of the mice in the model group were significantly higher than those in the blank group (*p* < 0.05). This indicates that the establishment of a diabetic mouse model was successful. Compared with the model group mice, the results showed that the administration of HIOP2-S for 21 days to diabetic mice significantly decreased (*p* < 0.05) the blood glucose level. Consequently, the HIOP1-S and HIOP2-S could elevate fasting blood glucose, and more importantly, HIOP2-S exhibited a stronger effect than HIOP1-S.

### 2.11. Effects of HIOP1-S and HIOP2-S on the Body Weights of STZ-Induced Diabetic Mice

Table 4 illustrates the effect of HIOP1-S and HIOP2-S on body weight. In the first week, due to the injection of STZ, the weight of the model group mice was significantly lower (*p* < 0.05) than in normal mice, in the next week, compared with the normal control group. Body weight of the animals in groups treated with HIOP1-S and HIOP2-S significantly (*p* < 0.05) decreased. However, there was no significant difference in body weight between the sample groups and the body weight of the model group. These results indicate that HIOP1-S and HIOP2-S have no side effects on mice.

## 3. Materials and Methods

### 3.1. Materials and Regents

The fruiting bodies of *Inonotus obliquus* were purchased from Greater Khingan Range, ground in a tight disintegrator (Micron Co. Ltd., Beijing, China), passed through a rough 40-mesh screen and stored at 4 °C during experiments. DEAE-52 column chromatography was purchased from the Pharmacia Chemical Co. (Trenton, NJ, USA) and Sephadex G-100 was purchased from Waters Chemical Co. (Milford, MA, USA). Monosaccharide standards, insulin, α-Glucosidase, p-nitrophenyl-α-d-glucopyranoside (pNPG) and STZ were purchased from Sigma-Aldrich Co. (St. Louis, MO, USA). Foetal bovine serum (FBS), Dulbecco’s Modified Eagle’s medium (DMEM), penicillin, streptomycin and phosphate-buffered saline (PBS) were purchased from Beijing Solarbio Science & Technology Co., Ltd. (Beijing, China). A glucose test kit was purchased from Shanghai Rongsheng Biotech Co., Ltd. (Shanghai, China). All other chemicals and solvents were of analytical grade.

### 3.2. Extraction, Isolation and Purification of Polysaccharides

The powdered *I. oblique* fruiting bodies were extracted with water at 30 times the volume at high temperature (90 °C) for 3 h and subsequently centrifuged at 4000 rpm for 20 min. Next, the supernatant was concentrated in a rotary evaporator under reduced pressure, and a three-fold volume of ethanol was added to the concentrate to precipitate the polysaccharides overnight at 4 °C. Finally, the precipitated was collected and dried at 50 °C. The crude polysaccharide was named HIOP.

HIOP was dissolved in distilled water (0.5%, *w*/*v*), filtered through a 0.45-μm Millipore filter, and subsequently the solution was loaded onto an anion-exchange DEAE-cellulose column (2.6 cm × 50 cm) equilibrated with distilled water. The column was eluted with distilled water and 0.2 mol/mL NaCl, respectively. The eluent was collected at a flow rate of 2.00 mL/min. Four millilitre fractions were collected in a single tube. The two collected HIOP fractions, denoted as HIOP1 (HIOP washed with distilled water) and HIOP2 (HIOP washed with 0.2 mol/L NaCl), were further fractionated by size-exclusion chromatography on a Sephadex G-100 column (2.6 cm × 70 cm) and eluted with deionized water. Each fraction was monitored using a phenol-sulphuric acid method for the determination of the carbohydrate content at 485 nm. Two fractions, denoted as HIOP1-S and HIOP2-S, were obtained and subsequently lyophilized.

### 3.3. Chemical Composition Analysis of HIOP1-S and HIOP2-S

The total carbohydrate content was measured using a phenol-sulphuric acid method with glucose as the standard [27]. The protein content was determined using the Bradford method [28], with bovine serum albumin as the standard. The uronic acid content was estimated according to Blumenkrantz & Asboe-Hansen [29]. Using galacturonic acid as the standard.

### 3.4. Homogeneity and Molecular Weight Analysis

The homogeneity and molecular weight distribution of HIOP1-S and HIOP2-S were determined using gel permeation chromatography (GPC) [30], as described above. A sample solution (20 µL) was injected and run with 0.1 mol/L NaNO_3_ buffer solutions at a flow rate of 1.0 mL/min at 30 °C. The peaks were detected using an Agilent 1206 system equipped with an ULtrahydrogel TM linear column (7.8 mm × 300 mm) and a Waters 2410 interferometric refractometer. The average MW (weight-average molecular weight) values of the polysaccharides were determined based on a standard curve produced using dextran T standards of known MW (T-180, T-2700, T-5250, T-9750, T-13050, T-36800, T-64650 and T-135350 Da).

### 3.5. Monosaccharide Composition Analysis

The monosaccharide composition of HIOP1-S and HIOP2-S polysaccharides were determined using a PMP-labelling procedure [31]. Polysaccharide samples (10 mg) were hydrolysed with 2 M TFA (trifluoroacetic acid) at 105 °C for 3 h in a sealed test tube. Subsequently, the excess TFA was removed by drying with the addition of methanol three times. The residue was re-dissolved in 3 mL of deionized water. Next, the polysaccharide hydrolysis solution (1 mL) was labelled with 1-phenyl-3-methyl-5-pyrazolone (PMP) through the addition of 600 µL 0.3 M NaOH and 600 μL of 0.5 M PMP methanol solution. The mixture was incubated at 70 °C for 2 h. The reaction product was neutralized with 600 µL of 0.3 M HCl solution and extracted three times with 2 mL chloroform. The aqueous phase was filtered through a 0.45-μm nylon membrane for HPLC analysis. The HPLC analysis was coupled to an UV detctor and a Agilent Eclipse XDB-C18 column (250 mm × 4.6 mm) matained at 30 °C. The column (250 mm × 4.6 mm) was eluted with a mobile phase consisting of 0.02 M phosphate buffer and acetonitrile (pH 6.7, 82:18, *v*/*v*) at 1 mL/min. Standard monosaccharides were determined using the same procedure. The monosaccharide content was calculated according to the calibration curve (peak area-concentration) of each monosaccharide standard.

### 3.6. Infrared Spectral Analysis (FT-IR)

The Nicolet Nexus FT-IR spectrometer (Thermo Nicolet Nexus, Waltham, MA, USA) was used to detect functional groups. HIOP1-S and HIOP2-S (2 mg) were separately mixed with dried KBr and pressed into a disk for subsequent analysis. The spectrum was recorded at a frequency range of 4000 to 400 cm^−1^ [32].

### 3.7. Nuclear Magnetic Resonance (NMR) Spectroscopy

The polysaccharide (36 mg) was deuterium-exchanged and dis- solved in 0.5 mL of D2O (99.8% D). The 1H-NMR spectra were measured at 25 °C with Brucker AVANCE600 NMR spectrometer (Beijing, China), with acetone as internal standard (d 31.50 ppm for carbon). 

### 3.8. Ultraviolet Spectrum Scanning 

Take a certain amount of the polysaccharide sample, dissolved in distilled water to a concentration of 0.1 mg/mL, and then measured in an ultraviolet scanner having a wavelength of 200–400 nm at 25 °C to obtain an ultraviolet scan.

### 3.9. Congo Red Test

The idea that the higher structure of the polysaccharide, especially the chain conformation, plays a greater role in the activity than the primary structure is well recognized by the scientific community, such as Schizophyllan which exhibits antitumor activity in the triple helix conformation, once the triple helix conformation becomes with irregular coils, anti-tumor activity disappears [33]. The biological activity of polysaccharides is not only based on the primary structure of polysaccharide molecules, but many physiological functions of polysaccharides need to be realized in liquid environment, so it is important to understand the superstructure conformation of polysaccharides in solution.

5 mg different polysaccharide were taken, add 2.0 mL distilled water and 2.0 mL Congo red reagent (80 μmol/L), 4 mol/L NaOH was added gradually, matained the concentration of the solution in the range of 0–0.05 mol/L, and then scanning by the Ultraviolet to measure the maximum absorption wavelength under each alkaline condition.

### 3.10. Assay of *α*-glucosidase Inhibitory Activity

The inhibitory effect of polysaccharide on α-glucosidase activity was determined as previously reported, with some modification [34]. First, 60 μL of 0.01 mol/L phosphate buffer (pH 6.8), 20 μL of α-glucosidase solution (2 U/mL), and 20 μL of 10 μg/mL polysaccharide sample solution were incubated in a 96-well microtiter plate at 37 °C for 10 min. After pre-incubation, 20 μL of 5 mmol/L pNPG was added and incubated for another 10 min. Finally, 30 μL of 0.1 mol/L Na_2_CO_3_ was added to terminate the reaction, and the absorbance at 405 nm was measured using a multimode microplate reader. Acarbose was used as the positive control and all the tests were conducted in triplicate. The α-glucosidase inhibition rates (*R*) were calculated according to the following formula:

R=[(A1−A2)−(A3−A4)](A1−A2)×100%

where: *A*_1_—the absorbance values of the blank; *A*_2_—the absorbance values of the blank control; *A*_3_—the absorbance values of the sample; *A*_4_—the absorbance values of the sample control.

### 3.11. Cytotoxicity Assay to HepG2 Cells

HepG2 cells were grown in Dulbecco’s Modified Eagle’s Media (DMEM), supplemented with 10% foetal bovine serum (FBS), and penicillin (100 U/mL)/streptomycin (100 μg/mL). The cells were maintained in an incubator at 37 °C and 5% CO_2_.

To access the cytotoxicity of the polysaccharides on the viability of HepG2 cells, a 3-(4,5-dimethylthiazol-2-yl)-2,5-diphenyltetrazolium bromide (MTT) assay was used to detect the cell viability after treatment with the polysaccharides at different concentrations [34]. After incubation, the cells were incubated with 20 μL of MTT solution (5 mg/mL) in the medium for 4 h at 37 °C. After incubation for 4 h, culture medium was removed and 150 µL of dimethyl sulphoxide (DMSO) was added into each well. The optical density (OD) at 570 nm was recorded with a spectrophotometer.

### 3.12. Glucose Consumption Assay

HepG2 cells were grown in DMEM supplemented with 10% FBS and penicillin (100 U/mL)/streptomycin at 37 °C. The cells were seeded onto 96-well plates with six wells left empty as blank wells. After the cells reached 80–90% confluence, the medium was replaced with DMEM supplemented with 1% FBS for 12 h. Two hours later, the medium was removed, and the same medium containing insulin (107 mmol/L), dried polysaccharides (10, 20, and 40 μg/mL, respectively) and metformin (10^−3^ mmol/L) was added to all wells, including the blank wells. After incubation for 24 h, the glucose concentrations in the cell supernatant were determined at 505 nm according to the instructions of the glucose test kit, and the glucose consumption rate was calculated based on the following formula [35]:

*R* (mmol/L) = (glucose concentrations of blank wells − glucose concentrations of cell plated wells)/glucose concentrations of blank wells

### 3.13. Animal Experiments

Male ICR (Institute of Cancer Research) mice (20 ± 2 g) were purchased from the Experimental Animal Center of the Chinese Academy of Military Sciences [License No. SCXK (Army) 2002-001]. After one week of adaptive feeding, fasting but only water for 12 h, a small dose of pre-cooled STZ solution was injected intraperitoneally with a single injection dose of 35 mg/kg, every 4 times a day for 3 days, during the intragastric administration of the mice, fresh water was replaced every day to ensure the fresh water supply; the litter was changed every day to ensure the clean environment of the mice; the temperature of the breeding room was kept at 22 ± 1 °C; the humidity was 50–65%. 48 h after the last injection of STZ, the mice were fasted for 6 h, the blood was taken from the tail vein and the fasting blood glucose was determined, when the blood glucose level was ≥12 mmol/L, we considered the model construction was successful. All animal handling procedures were carried out in strict accordance with the Chinese People’s Republic of China Laboratory Animal Use and Care Regulations and guidelines developed by the Laboratory Animal Center and approved by the Ethics Committee, and the permit number is KY110023.

The successful modeled mice were randomly divided into a model group (STZ induction), a positive control group (125 mg/kg metformin hydrochloride) and a polysaccharide treatment group (125 mg/kg, HIOP1-S, HIOP2-S), 12 mice in each group, and 12 healthy mice were used as the blank control group to ensure that the mice were given the same daily gavage time. The normal control group and the model group were given the same amount of sodium chloride solution. During the gavage, the mice were weighed weekly and the blood glucose level was measured.

### 3.14. Statistical Analysis

SPSS 17.0 software (IBM SPSS, Chicago, IL, USA) was used for the statistical analysis. The data are expressed as the means ± SD. *p* values of less than 0.05 or 0.01 were considered statistically significant.

## 4. Conclusions

In the present study, the water-extracted crude polysaccharide (HIOP) was fractionated through DEAE-52 cellulose and Sephadex G-100 column chromatography. Two major polysaccharide fractions (HIOP1-S and HIOP2-S) were obtained. The chemical and physical properties of these polysaccharides were determined using chemical methods, including GPC, IR, HPLC, and so on. The in vitro and vivo hypoglycaemic activities of the two major polysaccharides were also evaluated. The results indicated that HIOP1-S and HIOP2-S have high potential to act as a substitute for the standard oral hypoglycaemic agents metformin and acarbose, reflecting the high hypoglycaemic effects and safety of these polysaccharides, and HIOP1-S and HIOP2-S can be developed as potential new anti-diabetic drugs for further treatment. Further chemical and pharmacological investigations are needed to evaluate the mechanism underlying the hypoglycaemic actions of these compounds.

## Figures and Tables

**Table 1:** Monosaccharide composition and percentage.

Sample	HIOP1-S (%)	HIOP2-S (%)
Carbohydrate	82.73 ± 3.01 ^a^	84.62 ± 1.09 ^a^
Protein	1.79 ± 0.74 ^a^	1.93 ± 0.32 ^a^
Uronic acid	9.98 ± 1.34 ^a^	11.45 ± 0.98 ^a^
Mannose	1.75	9.714
Rhamnose	12.233	15.331
Glucose	29.673	49.881
Galactose	20.547	15.321
Xylose	2.386	4.675
Arabinose	15.786	5.078
Fucose	17.626	-

Means ± S.D.; ^a^ *p* > 0.05 HIOP1-S vs. HIOP2-S.

**Table 2:** The IC50 of H1OP1-S and H1OP2-S.

Polysaccharides	IC50 (µg/mL)
HIOP1-S	7.875
HIOP2-S	3.841
acarbose	2306.018

**Table 3:** Effect of HIOP1-S and HIOP2-S on the fasting blood glucose levels of Streptozotocin-induced diabetic mice.

Groups	Numbers	Dose (mg/kg bw)	Blood Glucose (mmol L^−1^)			
			0 day	7 days	14 days	21 days
Blank control	12	0	7.74 ± 5.30	8.72 ± 1.24	9.96 ± 1.8	9.70 ± 1.70
Model group	12	0	19.02 ± 7.05 *	22.81 ± 2.02	34.25 ± 1.94	19.50 ± 1.86
Met	12	125	19.95 ± 6.23 *	18.24 ± 3.95 **	25.92 ± 5.98 **	16.62 ± 3.2 **
HIOP1-S	12	4.5	18.24 ± 7.49 *	21.79 ± 5.02	28.04 ± 7.32	18.35 ± 3.02
HIOP2-S	12	4.5	18.53 ± 7.21 *	18.59 ± 7.69	28.25 ± 10.78	14.9 ± 5.26 **

Means ± S.D.; bw, body weight. * *p* < 0.05 vs. Normal control group. ** *p* < 0.05 vs. Diabetic control group.

**Table 4:** Effects of HIOP1-S and HIOP2-S on the body weights of STZ-induced diabetic mice.

Groups	Numbers	Dose (mg/kg)	bw/g			
			0 day	7 days	14 days	21 days
Blank control	12	0	24.89 ± 1.35	30.05 ± 2.87	32.23 ± 2.83	34.84 ± 2.87
Model group	12	0	23.41 ± 1.78	25.15 ± 1.73 *	27.51 ± 1.92 *	29.22 ± 2.55 *
Met	12	125	24.41 ± 1.63	26.75 ± 2.01 *	28.39 ± 2.15 *	30.25 ± 2.74 *
HIOP1-S	12	4.5	24.50 ± 2.39	27.58 ± 2.34 *	28.29 ± 2.38 *	28.41 ± 2.61 *
HIOP2-S	12	4.5	23.95 ± 1.69	25.59 ± 3.29 *	26.97 ± 2.75 *	28.31 ± 2.92 *

Mean ± S.D.; bw, body weight. * *p* < 0.05 vs. Normal control group.
